# Prevalence and genotype assessment of HPV in 73,697 females from Beijing, China

**DOI:** 10.3389/fpubh.2025.1623627

**Published:** 2025-09-05

**Authors:** Mingjian Bai, Yunxiang Li, Fucun Ma, Qian Gao, Zhiyong Lv, Yueming Xu, Jing Feng, Fengxian Fu, Guowei Liang

**Affiliations:** ^1^Department of Clinical Laboratory, Aerospace Center Hospital, Beijing, China; ^2^Peking University Fifth School of Clinical Medicine, Beijing, China; ^3^Department of Pathology, Aerospace Center Hospital, Beijing, China; ^4^Department of Literature and Science, University of Wisconsin-Madison, Madison, WI, United States; ^5^Department of Gynecology, Aerospace Center Hospital, Beijing, China

**Keywords:** human papillomavirus, prevalence, genotype, pathology, vaccination

## Abstract

**Background:**

The present study aimed to assess the prevalence and genotype distribution of Human papillomavirus (HPV) in Beijing females.

**Methods:**

A total of 73,697 subjects [both Department of Gynecology (*n* = 35,666) and Physical Examination center (*n* = 38,031)] were retrieved between June 2014 and December 2023. HPV testing was performed by Tellgenplex® HPV-27 DNA genotyping Test system.

**Results:**

An overall HPV infection rate of 17.28% (12,736/73,697) over the past decade (2014 to 2023), with a significant decrease in annual incidence (*p* < 0.001). HPV-52, 16, and 58 were predominant in high-risk types, while HPV-61, 81, and 43 were the most prevalent in low-risk HPV types. Age-specific patterns revealed bimodal infection peaks in individuals under 21 and between 51 ~ 56 years. Furthermore, a significant increase in HPV infection rates was observed with the progression of pathological severity (*p* < 0.001).

**Conclusion:**

This study emphasizes the importance of HPV infection rate from real word and developing vaccines specifically for the Chinese population. In the future, it is necessary to design more suitable vaccines based on Chinese HPV type ranking, especially for HR-HPV (HPV-39, HPV-53, and HPV-56) and LR-HPV (HPV-43, HPV-61, and HPV-81).

## 1 Introduction

Cervical cancer continues to rank among the top gynecologic cancers worldwide. According to current data, it is ranked as the 4th most common cancer among women worldwide (1). In 2022, cervical cancer was the eighth most prevalent cancer, with 661,021 new cases reported globally (2). In China, cervical cancer ranked sixth among malignant tumors in 2022, with 150,700 incident cases (3). Persistent infection by high-risk human papillomavirus (HR-HPV) served as the major risk factors (4, 5). While low-risk (LR-HPV) infection has been associated with conditions like plantar warts, genital warts, and hand lesions (6, 7).

Vaccination is a primary preventive measure against cervical cancer (8). The World Health Organization (WHO) aims to eliminate cervical cancer by 2030 through the 90–70-90 strategy (9). The bivalent vaccine has shown 90% efficacy in protecting against HPV-16 and HPV-18 (5). The quadrivalent HPV vaccine showed an 88% reduction in cervical cancer incidence among those vaccinated before age 17 (10). Additionally, the nine-valent HPV vaccine could potentially provide broader coverage and prevent 90% of cervical cancer cases (11). By now, the available HPV vaccines in China were mainly invented using epidemiological data only from western countries (12). However, the prevalence of HPV infection and genotype distribution varies between countries and regions (13). Which highlighting the importance of analyzing baseline HPV infection data in different regions to evaluate vaccine effectiveness and adjust cancer prevention strategies.

Through literature search, in China, most studies on HPV infection rates mainly focused on the gynecological patients (14, 15), while the HPV infection data in healthy individuals is relatively lacking, as a consequence, we believe that this will significantly overestimate HPV infection rates in China. Similarly, we found that HPV infection survey in the Beijing also mostly come from gynecological patients (4, 16), which also be confront of the selection bias problem. Therefore, it is of great necessary to conduct a new epidemiological survey on HPV infection in Beijing, which should include both gynecological and health personnel. Secondary prevention measure for Cervical cancer including HPV screening and cytological examinations (8). Most screening and diagnostic efforts are directed toward the early identification of HR-HPV and Papanicolaou (Pap) smears (1).

The present study aimed to achieve the following work. First, the prevalence and genotype distribution of HPV will be surveyed, importantly, all participants not only from Department of Gynecology but also Physical Examination (PE) center, therefore, the prevalence estimation about HPV is closer to the real world in general population. Second, to compare of HPV positive rates in different pathological grades based on ThinPrep cytologic test (TCT) results.

## 2 Methods

### 2.1 Patients and sample collection

**Figure 1:**
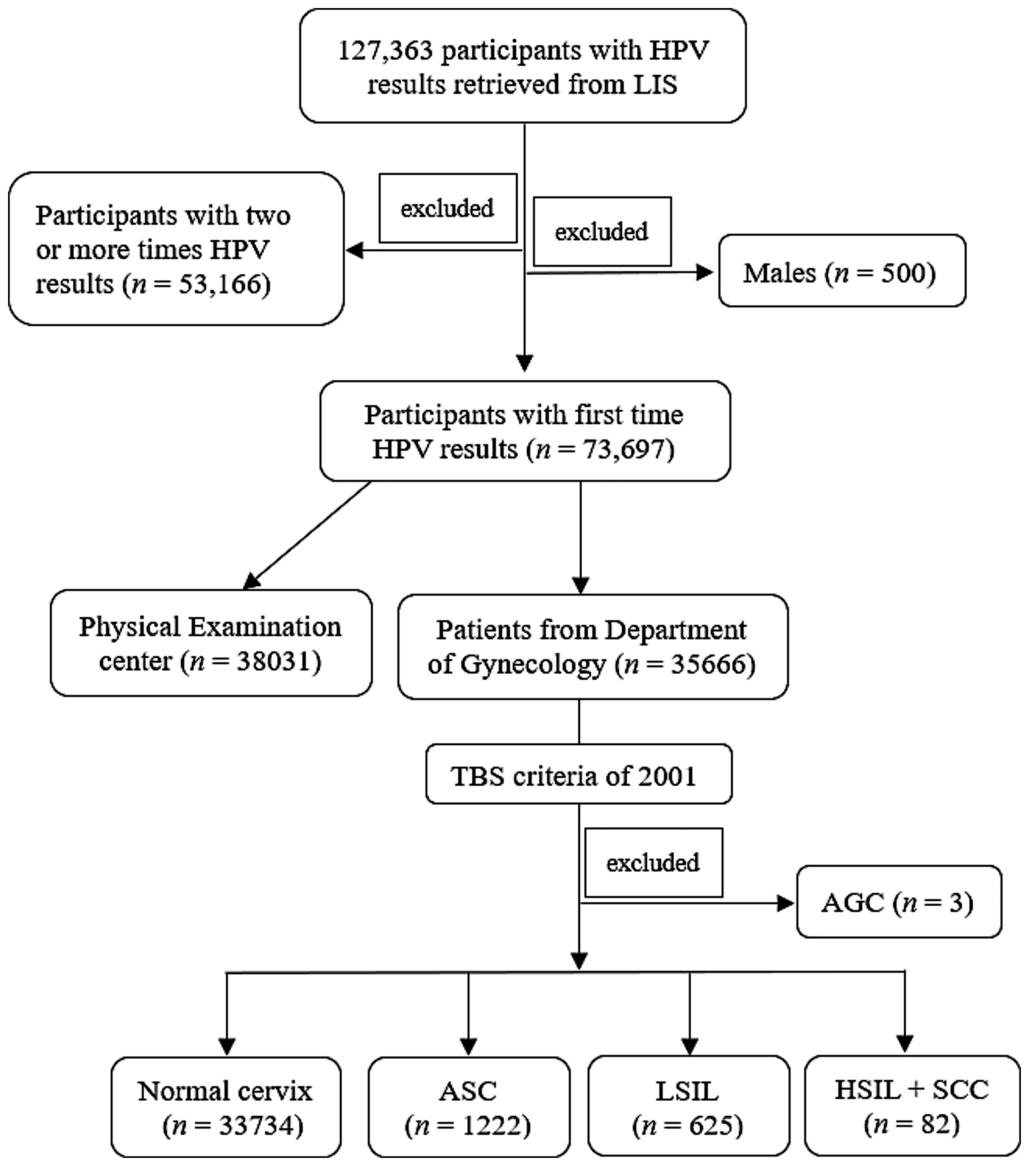
Study schematic. LIS, laboratory information system; TBS, The Bethesda System; AGC, atypical glandular cells; ASC, atypical squamous cells; LSIL, low-grade squamous intraepithelial lesion; HSIL, high-grade squamous intraepithelial lesion; SCC, squamous cell carcinoma.

This retrospective study was approved by the Institutional Review Board (IRB) of Aerospace Center Hospital. A total of 127,363 HPV results were retrospectively retrieved from laboratory information system (LIS) of Aerospace Center Hospital (monocentric) between June 2014 and December 2023. The first time HPV detection of all participants in our center were included, while the second or more HPV tests for the same participants were excluded (*n* = 53,166). Besides, the male subjects were also excluded (*n* = 500). Finally, a total of 73,697 female subjects from both the Department of Gynecology (*n* = 35,666) and PE center (*n* = 38,031) were included. Patients with menstrual problems, abnormal leucorrhea, etc. visited Gynecology Department, while participants only for health check-up were admitted to PE center (Figure 1).

To ensure the accuracy of the test results, all participants were advised to refrain from using vaginal medications or engaging in sexual intercourse within 3 days prior to testing. During specimen collection, clinical physicians use a specialized cervical brush to collect exfoliated cells, which were then placed into an elution tube containing cell preservation solution and sealed for storage. For gynecological patients, two sets of specimens were routinely collected, one for HPV testing, and another set for TCT.

### 2.2 HPV-DNA genotype testing

HPV testing was performed by Tellgenplex® HPV27 DNA genotyping Test system (Tellgen Corporation, Shanghai). The experimental protocol includes DNA extraction, multiple PCR amplification, bead-coated hybridization, and digital signal processing (Luminex 200TM, Thermo Fisher) (12). The totaling detectable HPV types was 27, including LR-HPV types (6, 11, 40, 42, 43, 44, 55, 61, 81, 83) and HR-HPV types (16, 18, 26, 31, 33, 35, 39, 45, 51, 52, 53, 56, 58, 59, 66, 68, 82). The mixed HPV infection means co-infection with both LR-HPV and HR-HPV.

### 2.3 TCT determination

According to The Bethesda System (TBS) of 2001 (17), TCT results were mainly divided into two major parts: Negative for Intraepithelial Lesion or Malignancy and Epithelial Cell Abnormalities, subsequently, the abnormal part was further classified according to the TBS criteria. In the present research, there were only three patients with atypical glandular cells (AGC), who were then excluded when performing TCT analysis. Finally, TCT was divided into five categories: (a) Negative for Intraepithelial Lesion or Malignancy; (b) Atypical squamous cells (ASC) of undetermined significance (ASC-US) or cannot exclude HSIL (ASC-H); (c) low-grade squamous intraepithelial lesion (LSIL), encompassing cervical intraepithelial neoplasia (CIN) 1; (d) High-grade squamous intraepithelial lesion (HSIL), encompassing CIN II and CIN III, and (e) Squamous cell carcinoma (SCC). Due to the small number of HSIL and SCC subgroup, which were then incorporated into the HSIL+SCC group (Figure 1). All TCT results were independently reviewed by at least two experienced pathologists. In case of discordance between the two pathologists’ interpretations, the joint review will be performed to resolved discrepancies.

### 2.4 Statistical analysis

Statistical analyses were conducted using *SPSS* software (version 20.0; IBM Corporation, Armonk, NY, USA). *Chi-square* tests or *Fisher’s* exact tests were applied when comparing HPV infection positivity rates across different years or age groups, as appropriate. The linear-by-linear association test and gamma value were utilized to evaluate the changes in HPV prevalence across the years and age groups. *GraphPad Prism* 16 was employed for data visualization, with cumulative frequencies used for calculating HPV types. Statistical significance was set at *p* < 0.05.

## 3 Results

### 3.1 The overall prevalence of HPV infection

For the included 73,697 participants, the average age is 42.88 ± 12.88 years. There were 12,736 female subjects confirmed to be infected by HPV with the infection rate of 17.28% (95% CI: 16.89–17.67%). The single and multiple HPV infection rate were 12.65% (95% CI: 12.39–12.91%) (9,326/73697) and 4.63% (95% CI: 4.47–4.79%) (3,410/73697), respectively (Table 1).

**Table 1:** Explicated HPV infection number among the 73,697 participants.

Number of HPV types (*n*)	Frequency (*n*)	Percent (%)
0	60,961	82.72
1	9,326	12.65
2	2,500	3.39
3	669	0.91
4	178	0.24
5	49	0.07
6	10	0.01
7	3	0.00
8	1	0.00

**Figure 2:**
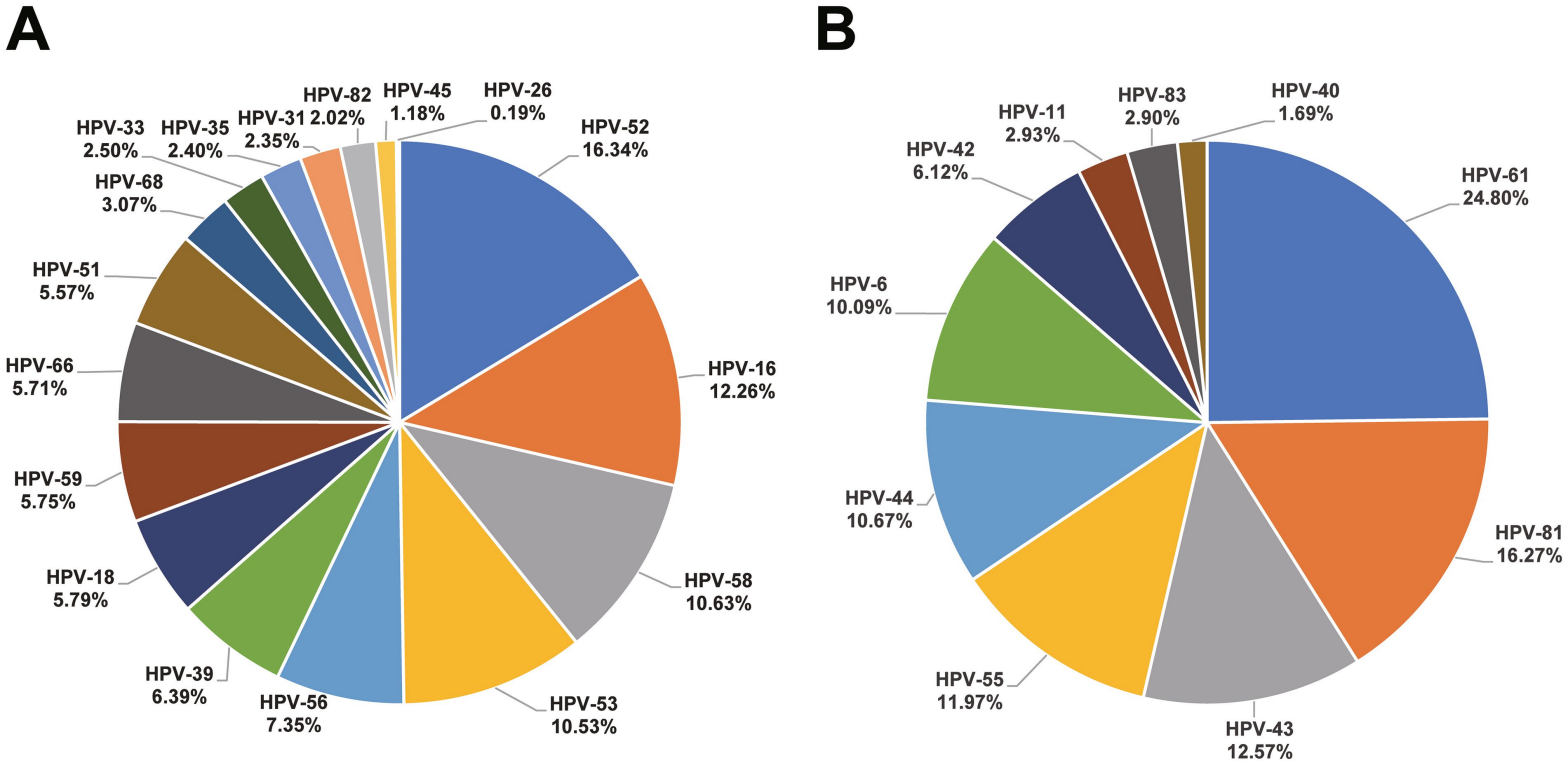
Proportion of HPV types in HR-HPV **(A)** and LR-HPV **(B)**.

A total of positive 17,379 HPV results (including 12,233 HR-HPV and 5,146 LR-HPV) were detected among these 12,736 HPV infected subjects. HPV-52 (16.34%), HPV-16 (12.26%), and HPV-58 (10.63%) were the most common in HR-HPV, as for LR-HPV, HPV-61 (24.80%), HPV-81 (16.27%), and HPV-43 (12.57%) were predominant. The distribution of HPV types is depicted in Figure 2. Additionally, in the HR-HPV group, the proportion of single infections (7,910/9852, 80.29%) exceeds that of multiple infections (infected by two or more HPV types) (1942/9852, 19.71%). As for LR-HPV group, single infections (4,032/4561, 88.40%) surpass multiple infections in the same way (529/4561, 11.60%). A total of 1,677 subjects infected were infected with both HR-HPV and LR-HPV, the mixed infection rate was 2.28%.

Separately, in the PE group, the infection rate was 13.17% (95% CI: 12.81–13.53%) (5,007/38031), while in the patient group, it was 21.67% (95% CI: 21.19–22.15%) (7,729/35666), the HPV infection rate between the two groups reached a statistical significance (*χ*^2^ = 931.317, *p* < 0.001).

### 3.2 Distribution of HPV infection rates among different years

**Figure 3:**
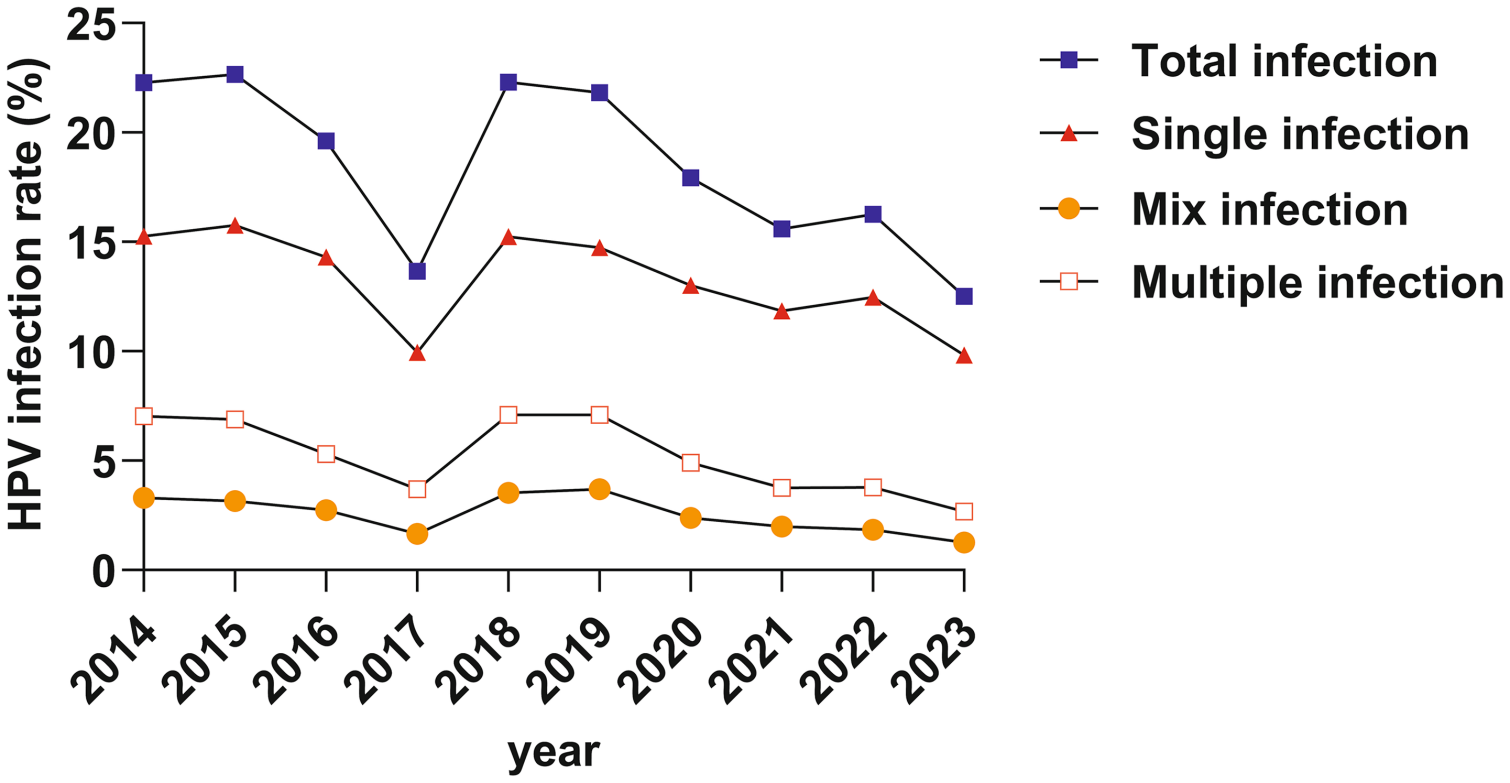
Different permutation of HPV infection rate over the past decade.

The infection rates varied significantly over the years (all *p* < 0.001). Over the past decade, there has been a downward trend (All gamma values are negative) in total infection, single infection, multiple infection, and mixed infection (infected by both LR-HPV and HR-HPV) (all *p* < 0.001) (Table 2; Figure 3).

**Table 2:** The HPV infection rate of different combination over the past decade.

Infection combination	2014	2015	2016	2017	2018	2019	2020	2021	2022	2023	*χ* ^2^	*p*	*χ* ^2^ (liner by liner)	*p* (liner by liner)	Gamma
Total cases	2,602	4,884	4,724	4,705	4,742	3,930	12,989	13,890	11,102	10,129					
Single infection	397 (15.26%)	770 (15.77%)	676 (14.31%)	468 (9.95%)	722 (15.23%)	579 (14.73%)	1,691 (13.02%)	1,645 (11.84%)	1,383 (12.46%)	995 (9.82%)	229.041	0.001	107.51	0.001	−0.078
Multiple infection	183 (7.03%)	336 (6.88%)	251 (5.31%)	174 (3.7%)	336 (7.09%)	279 (7.1%)	638 (4.91%)	521 (3.75%)	421 (3.79%)	271 (2.68%)	355.415	0.001	210.14	0.001	−0.175
Mix infection	86 (3.31%)	154 (3.15%)	129 (2.73%)	78 (1.66%)	168 (3.54%)	145 (3.69%)	309 (2.38%)	275 (1.98%)	204 (1.84%)	129 (1.27%)	172.772	0.001	88.468	0.001	−0.164
Total infection	580 (22.29%)	1,106 (22.65%)	927 (19.62%)	642 (13.65%)	1,058 (22.31%)	858 (21.83%)	2,329 (17.93%)	2,166 (15.59%)	1804 (16.25%)	1,266 (12.5%)	548.319	0.001	294.87	0.001	−0.115

### 3.3 Prevalence of HPV infection in different age groups

**Figure 4:**
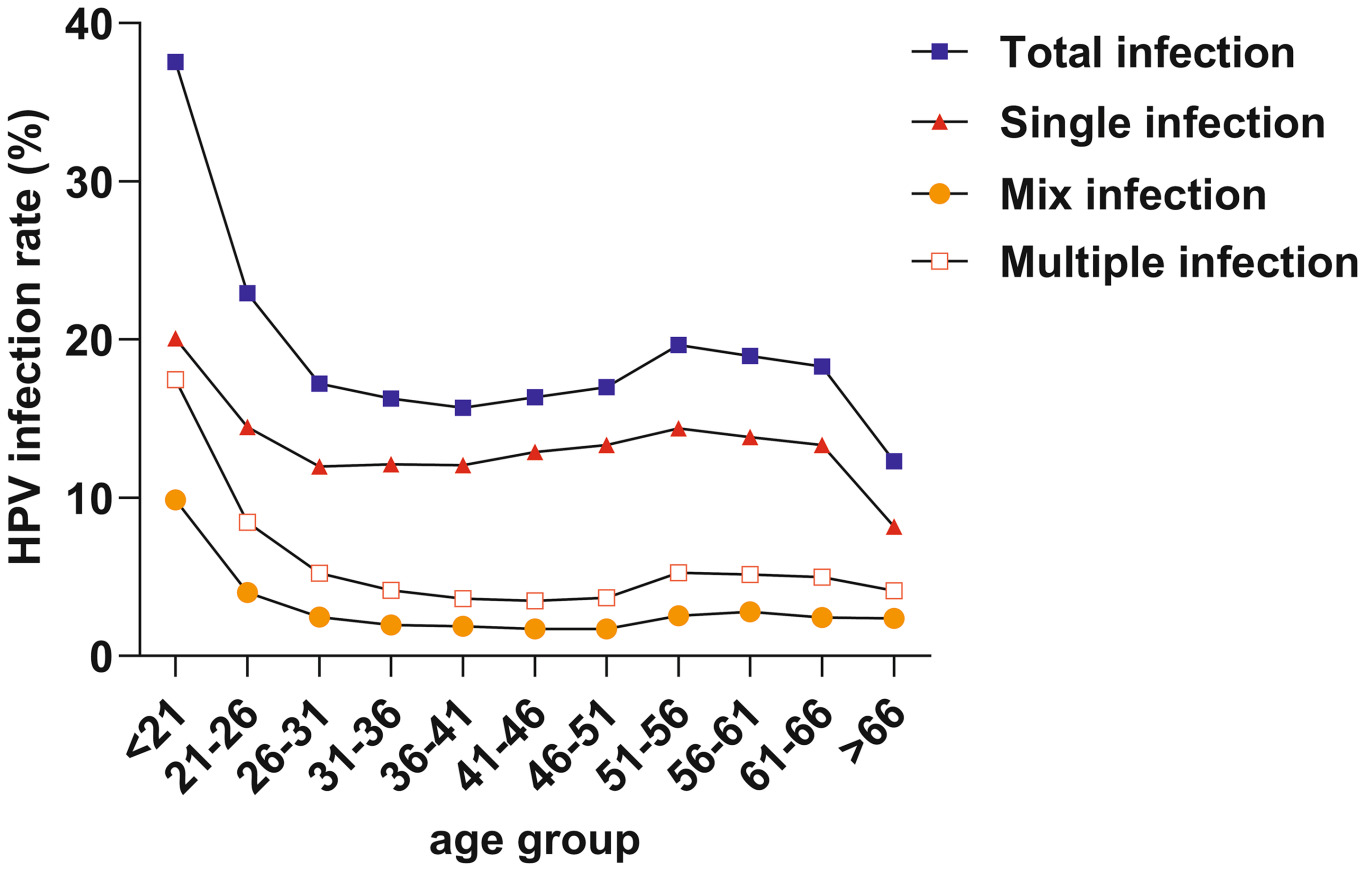
HPV infection bimodal distribution in different age (years) groups.

We divided the ages into 11 subgroups, starting from 21 years old, with each group spanning 5 years. The total infection rate exhibited a bimodal distribution, with the first peak at < 21 years (37.55%) and a gradual decline followed by a second peak at 51–56 years (19.64%). Furthermore, trends in single infection, multiple infection, and mixed infection mirrored those of total infection, with the first peaks at < 21 years old and a second peak at 51–56 years old (Table 3; Figure 4).

**Table 3:** Prevalence of HPV infection rates among different age groups.

Age group (years)	Cases	Single infection	Multiple infection	Mix infection	Total infection
< 21	538	108 (20.07%)	94 (17.47%)	53 (9.85%)	202 (37.55%)
21–26	4,132	598 (14.47%)	349 (8.45%)	166 (4.02%)	947 (22.92%)
26–31	10,589	1,269 (11.98%)	554 (5.23%)	259 (2.45%)	1823 (17.22%)
31–36	12,277	1,486 (12.1%)	510 (4.15%)	241 (1.96%)	1996 (16.26%)
36–41	11,007	1,326 (12.05%)	400 (3.63%)	205 (1.86%)	1726 (15.68%)
41–46	8,987	1,158 (12.89%)	312 (3.47%)	153 (1.7%)	1,470 (16.36%)
46–51	8,046	1,072 (13.32%)	296 (3.68%)	137 (1.7%)	1,368 (17.0%)
51–56	6,419	924 (14.39%)	337 (5.25%)	164 (2.55%)	1,261 (19.64%)
56–61	4,776	660 (13.82%)	246 (5.15%)	133 (2.78%)	906 (18.97%)
61–66	3,076	410 (13.33%)	153 (4.97%)	75 (2.44%)	563 (18.3%)
> 66	3,850	315 (8.18%)	159 (4.13%)	91 (2.36%)	474 (12.31%)

### 3.4 Prevalence of HPV infection rate among different TCT groups

The total HPV infection rate among Normal cervix, ASC, LSIL, and HSIL+SCC subgroups were 19.39% (6,541/33734), 51.88% (634/1222), 77.12% (482/625), and 87.80% (72/82), respectively (*χ*^2^ = 2103.703, *p* < 0.001), the linear-by-linear association test also disappeared an evident rising trend (gamma = 0.737). The single and multiple HPV infection rates across these cytological classifications also demonstrated analogous statistical findings (Table 4).

**Table 4:** HPV infection rates among the four TCT groups.

Type	Normal cervix (*n* = 33,734)	ASC (*n* = 1,222)	LSIL (*n* = 625)	HSIL+ SCC (*n* = 82)	*χ* ^2^	*p*	*χ* ^2^ (liner by liner)	*p* (liner by liner)	Gamma
Total infection	6,541 (19.39%)	634 (51.88%)	482 (77.12%)	72 (87.80%)	2103.703	0.001	2078.361	0.001	0.737
Single infection	4,683 (13.88%)	407 (33.31%)	270 (43.20%)	46 (56.10%)	844.654	0.001	828.054	0.001	0.571
Multiple infection	1858 (5.51%)	227 (18.58%)	212 (33.92%)	26 (31.71%)	1204.415	0.001	1176.353	0.001	0.683

## 4 Discussion

This retrospective study, investigated the overall HPV infection rate in Beijing, with an observed decline in annual incidence rates. Besides, the age distribution of HPV infections exhibits a bimodal pattern, and HPV infection rates showed an increasing trend with the severity of pathological classification. Importantly, the current nine-valent vaccine does not cover all the relatively high proportion HPV types in Beijing females.

Over the past decade, the average HPV infection rate was 17.28% in present study. Comparatively, data from other regions and central studies in China differed (4, 14, 18–20): the HPV detection positive rate ranged from 15.54% (21) to 45.57% (22). The variation can be attributed to several factors. Firstly, the majority of studies only included the gynecological outpatient or inpatient individuals, where infection rates are higher compared to the general population. Therefore, it cannot truly reflect the HPV infection rate among Chinese women in the real world. Our study included both gynecological patients and individuals undergoing routine health checks, similar to the study in Nanning, which also included both outpatient and health examination participants (20), providing a more accurate reflection of HPV infection rates among Chinese women in Beijing. Secondly, different economic conditions, health infrastructure, and vaccine uptake might also contribute to the variability in HPV prevalence, as observed in comparisons across different cities in China (23). The last reason cannot be ignored is that different testing reagents may lead to different HPV positivity rates. Our study utilized reagents that detected 27 types of HPV, suggesting that the more types tested, the higher the HPV positivity rate increases. Thereby, we suggest that all future literature must report the number of determined HPV types. Due to the variation in HPV infection rates across different regions worldwide, there is an urgent need to carried out a nationwide epidemiological investigation of HPV infection rate in China.

The most frequently occurring HR-HPV types in our study were HPV-52, HPV-16, and HPV-58, similar to data from Northern China (4), Sichuan (24), and Nanning (20). The prominence of HPV-52 in Beijing aligns with previous study (4), although not as strongly associated with cervical cancer as HPV-16 and HPV-18, HPV-52 is highly related to cervical lesions, emphasizing the importance of promoting the nine-valent vaccine in Beijing. However, HPV-18 (rank 9, 0.96%) did not represent a high proportion, differing from descriptions in some literature from the Asian region (rank 2, 1.4%) (25). Besides, the proportion of several HR-HPV (HPV-39, HPV-53, and HPV-56) are relatively high. However, the existing nine-valent HPV vaccines do not cover the relatively high-proportion HPV types listed above. As for LR-HPV, the proportion of HPV-43, HPV-61, and HPV-81 were significantly high than that of HPV-6 and HPV-11, unfortunately, these high-proportion types are also not included in the HPV vaccine by now. The above findings might have important implications for national vaccine strategy in China, such as the future vaccine formulation or expansion of genotype coverage domestic vaccines. It is highly necessary to develop vaccines specifically tailored for the Chinese population based on this epidemiological data.

Over the years, there has been a declining trend in HPV infection rates. This trend reflects the control of HPV transmission due to increased vaccine coverage and public health awareness. This trend is consistent with results from Southern China (18), but inconsistent with the rising trend observed in Guangzhou (15). This inconsistency may be attributed to Beijing’s advantages in vaccination and healthcare.

A bimodal distribution phenomenon in age groups has been observed in present research, which peaks in the < 21 and 51 ~ 56 years age groups, consistent with other studies (4, 18, 23). Moreover, a declining trend was observed in all age groups except those over 66 years, which may be attributed to the health conditions and public awareness. It is possible that older adults women face challenges in clearing prior infections and are at higher risk of new infections (26). The occurrence of bimodal peaks may be attributed to immature immune protection and high-risk behaviors in younger individuals (27), leading to a higher likelihood of progression to LSIL. In menopausal women, significant hormonal changes may alter the immune system, increasing susceptibility to HPV infections and reducing HPV clearance rates (26). A cervical cancer modeling study indicated that screening every 3 years for women aged 25–69 was associated with a lifetime risk of cervical cancer of 1 in 532 (28). In contrast, for unvaccinated and unscreened women, the lifetime risk of cervical cancer was 1 in 45. These results underscore the importance of government initiatives to promote HPV vaccination prior to sexual debut in young women and advocate for HPV screening in menopausal women.

As the primary cause of cervical lesions, HPV infection is almost universally present in patients with any cervical cancer pathology (29). The present study revealed a notable escalation in HPV infection rates concomitant with the progression of pathological severity, consistent with the results of a former systematic review (30), which reported overall infection rates of 59.6% for CIN I and 84.8% for CIN II+. Similarly, a meta-analysis in 2020 (31) indicated HPV infection rates of 79.56% for CIN I and 87.00% for CIN II/III. Besides, Previous research has reported that HPV-52, HPV-16, and HPV-58 was the top three prevalent types in China for CIN (32), which is consisted with our study.

The study is subject to limitations. First, it is a single-center retrospective study, which may affect generalizability to the broader Chinese population. Second, although we attempted to investigate the prevalence of HPV in the real world, the proportion of participants from the PE center and the Gynecological Department is close to 1:1, underestimating the proportion of the healthy population. Third, vaccination status was not obtained in the 73,697 female subjects, therefore, we could not assess the protection effectiveness of HPV vaccine in present study.

To conclude, present study investigated the prevalence and genotypes distribution of different HPV types from both healthy populations and Gynecological patients. The overall HPV infection rate has been decreasing year by year, appeared as a pronounced bimodal phenomenon grouped by age, and showed a significant upward trend with the progression of pathological severity. In the future, it is necessary to design more suitable vaccines based on Chinese HPV type ranking, especially for HR-HPV (HPV-39, HPV-53, and HPV-56) and LR-HPV (HPV-43, HPV-61, and HPV-81).

## Data Availability

All relevant data is contained within the article. The original contributions presented in the study are included in the article, further inquiries can be directed to the corresponding authors.
